# Effects of stress or infection on rat behavior show robust reversals due to environmental disturbance

**DOI:** 10.12688/f1000research.13171.2

**Published:** 2018-01-16

**Authors:** Samira Abdulai-Saiku, Akshaya Hegde, Ajai Vyas, Rupshi Mitra

**Affiliations:** 1 School of Biological Sciences, Nanyang Technological University, Singapore, 637551, Singapore

**Keywords:** anxiety, fear, construction, housing environment, replicability

## Abstract

**Background:** The behavior of animals is intricately linked to the environment; a relationship that is often studied in laboratory conditions by using environmental perturbations to study biological mechanisms underlying the behavioral change.

**Methods:** This study pertains to two such well-studied and well-replicated perturbations, i.e., stress-induced anxiogenesis and *Toxoplasma* *gondii* -induced loss of innate fear. Here, we demonstrate that behavioral outcomes of these experimental manipulations are contingent upon the ambient quality of the wider environment where animal facilities are situated.

**Results:** During late 2014 and early 2015, a building construction project started adjacent to our animal facility. During this phase, we observed that maternal separation stress caused anxiolysis, rather than historically observed anxiogenesis, in laboratory rats. We also found that *Toxoplasma gondii* infection caused an increase, rather than historically observed decrease, in innate aversion to predator odors in rats.

**Conclusion:** These observations suggest that effects of stress and *Toxoplasma gondii* are dependent on variables in the environment that often go unreported in the published literature.

## Introduction

Multiple laboratories have reported that stress causes anxiogenesis in rats ^1– 4^. Similarly, well-replicated studies indicate that infection of rats with protozoan *Toxoplasma gondii* reduces innate aversion to predator odor ^5– 11^. Effects of *Toxoplasma gondii* infection on fear are not absolute. Rather effects of the infection on aversion follow a non-monotonous function roughly resembling an inverted-U ^12^. Similarly, effects of stress on anxiety are also open to environmental modifications. Anxiety induced by stress can be reliably prevented if housing conditions of animals are changed ^1, 13^ or if animals have opportunity of voluntary exercise ^14– 16^. These observations suggest that effects of both the infection and stress on animal behavior are responsive to environmental modifications. In this backdrop, this report describes our serendipitous observations that the direction for both behavioral changes is intricately dependent on the broader environment where animal facilities are situated.

The primary aim of our experiments was to study proximate mechanisms of anxiogenesis and innate aversion in rats. We used routine paradigms of maternal separation and *Toxoplasma gondii* infection that cause anxiogenesis and loss of innate aversion, respectively. However, construction of a building was initiated during the experiment adjacent to the animal holding facility. Results from this quasi-experimental change provided us with an unplanned opportunity to study the effects of change in environment on rat anxiety and defensive behaviors.

## Methods

### Animals

Adult male and female Wistar Han rats (7 to 8 weeks at the start of the experiments) were procured from InVivos, Singapore. Rats were housed in groups of two per cage (males and females were housed separately) with ad libitum access to food and water (24–26°C; 60–70% relative humidity; 12h light-dark cycle with lights on at 0700h). For all tests, animals were allocated to groups in a random manner. Experiments were conducted by SA-S and AHN who were blind to group allocations. Analysis was conducted by AV who was also blind to group allocations. All procedures were approved by the Institutional Animal Care and Use Committee of the Nanyang Technology University. All efforts were made to ameliorate any suffering of animals. None of our procedure involved induction of sustained pain requiring pharmacological interventions. Animals were daily observed to confirm lack of sickness related behaviors and weighed weekly. The behavior tests do not involve any use of shock or other painful stimuli. The dose of parasites used in this study does not result in weight loss or sickness behavior in this strain of rats.

At the end of all experiments, animals used in the Toxoplasma infection paradigm were sacrificed by decapitation and their brains were removed and flash frozen. In the case of the stress paradigm, animals were sacrificed by cardiac perfusion using cold phosphate buffered saline (PBS) followed by cold 4% paraformaldehyde.

### *Toxoplasma gondii* infection and quantification of aversion to cat odor

Female rats were either injected with tachyzoites of type 2 Prugniaud strain of *Toxoplasma gondii* (5x10 ^6^ tachyzoites in 500 µl phosphate buffered saline, *i.p.*;) or mock injected with the buffer alone between 2pm and 4pm. Parasites needed for the infection were maintained *in vitro* in human foreskin fibroblast cultures and were harvested using syringe lysis. Behavioral experiments were conducted seven weeks post-infection; a time-window consistent with chronic phase of the infection. *Toxoplasma gondii* infection did not cause significant change in body weight of animals (179.1 ± 4.708, n = 8 for uninfected; 183.1 ± 2.706, n = 7 for infected; *p* = 0.5, independent sample t-test).

Aversion to cat odor was quantified in two different manners. For each run of the experiment, there was one uninfected group and one *Toxoplasma*-infected group. Fifteen animals were used in total for experiment 1 (8 uninfected, 7 infected) and nineteen animals were used in total for experiment 2 (10 uninfected, 9 infected).

**Figure 1.**
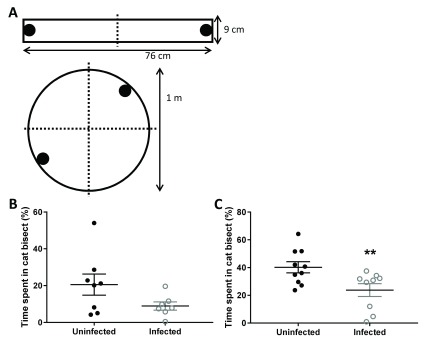
*Toxoplasma gondii*-infected female rats showed increased aversion to bobcat odor in two sequential experiments. Panel **A** depicts schematics of test arenas used in the experiments later depicted in panels **B** and **C**. Ordinate for panel **B** and panel **C** depicts time spent by female rats chronically infected with *Toxoplasma gondii* near bobcat odor. Line graphs depict mean and standard error of the mean for control (black) and infected (gray) female rats. *, *p* < 0.05; unpaired two-tailed Student’s t-test (n = 8 uninfected and 7 infected animals for panel **A**; and 10 uninfected and 9 infected animals for panel **B**).

Aversion was first quantified in a rectangular arena with two opposite and identical arms ( Figure 1A; 76 x 9 cm each), separated by a central part (9 x 9 cm in size; white Perspex). Animals were habituated to the arena for three consecutive days for 20 minutes each day. On the subsequent day, cat odors were presented in one bisect of the maze (1 ml each; bobcat urine from Maine Outdoor Solutions, USA). Animals were placed in the center of the maze and exploration time in both bisects of the arena was measured for 20 minutes. Trials were video recorded with offline analysis conducted using AnyMaze (Stoelting, USA). In this batch of animals, each received 500 µl of buffered saline intraperitoneally thirty minutes before the behavioral test.

Aversion to cat odor was also quantified in a circular arena ( Figure 1A; diameter = 1 m) that was arbitrally divided into four quadrants. Animals were habituated to the arena for three consecutive days for 20 minutes each day. On the subsequent day, cat odor, vanilla essence, water and the bedding from the animal’s home cage were presented in each quadrant of the maze. Animals were placed in the center of the maze and exploration time in all quadrants of the arena was measured for 20 minutes. Trials were video recorded with offline analysis conducted using AnyMaze (Stoelting, USA).

### Stress paradigm and quantification of anxiety

Eight week old breeders obtained from InVivos were allowed to acclimatize for at least 5 days before setting up breeding pairs (one male and one female per cage). Breeding cages were changed once a week as per normal, but with gentle handling of female, in case of pregnancy. Once pregnancy was certain (approx. 2 weeks), male was removed. 19 days after breeding pairs were set up (or if visually heavily pregnant), cages were checked daily for litters. Day of birth is assigned P0.

Maternal separation was used as the stress model (P2-P14, daily). 16 animals were used in total; 8 stressed, 8 unstressed. On each of these days, the dam was removed from the cage and placed in a new cage with unsoiled bedding. Pups were then retrieved into another cage with unsoiled bedding, transported to a separate room and put on a heating pad for three hours every morning. At the end of the separation period, pups and then dam were sequentially returned to the original soiled cage. Also, soiled bedding was changed on postnatal day 2, 9 and 14; by returning pups to a clean cage that had been supplemented with a scoop of soiled bedding and nesting material from the original cage. This practice was repeated on postnatal day 18 if the bedding was considered significantly soiled in case of large litter sizes. Pups were weaned on postnatal day 21. Anxiety was quantified when the male pups reached adulthood (7–8 weeks of age). Anxiety was measured using home cage emergence assay (adapted from 12) and elevated plus-maze ^13^.

In the home cage emergence assay, a rat placed in its home cage was transported to a well-lit room and habituated for five minutes. The cage was then left open by removal of the lid. The rat was offered a possibility of emerging from the home cage through a wire grid placed within the cage. The latency of emergence was recorded. Emergence was defined when all four limbs of the rat were placed on the grid. Trials were terminated at the emergence or at five minutes, whichever occurred earlier. Trials were video recorded and scored manually.

The elevated plus-maze consisted of a plus-shaped arena with two open (75 × 11cm, 1cm wall, 3–4 lux illumination) and two enclosed arms (75 × 11 cm, 26 cm wall). The arena was elevated to a height of 60 cm above the ground. The animal was placed at the center at the start of the trial. Exploration in open and enclosed arms was quantified for five minutes each.

All experiments for the stress paradigm were done using two groups of mice: stressed and unstressed.

### Statistical analysis

The probability of type 1 error was calculated using unpaired two-tailed Student’s t-test. The standardized effect size was calculated using Cohen’s *d* ^14^; with values above the magnitude of 0.8 interpreted as being of robust scale. Negative *d* values correspond to the comparisons where mean of experimental treatment was greater than that of respective controls. Mean inter-group difference was also calculated with 95% confidence intervals. Data is graphically presented as mean and standard error of the mean (SEM), along with individual values for each animal for each endpoint. Number of animals in each experimental group is noted in the figures. All statistical analysis was conducted using Graphpad Prism.

## Results

### *Toxoplasma gondii* infection increased aversion to cat odor

In the first set of animals, aversion to cat odor was quantified as percentage time in bisect containing cat odor relative to total trial duration. Rabbit odor was placed in the opposing bisect as a novel non-predator odor. Inter-group differences did not reach pre-determined threshold for statistical significance ( Figure 1B; t _13_ = 1.78, *p* = 0.098). Despite the lack of sufficiently low type 1 error, the effect on mean was of robust magnitude (Cohen’s *d* = 0.949; Δ = -11.61% with 95% confidence intervals -25.68 to 2.46%). The maximum of animals from the infected group was below the median of the uninfected animals. The robust effect size and the observation that infected mean was lower than uninfected animals in contrast to the multitude of published studies, led us to plan a further experiment to increase the statistical power.

In this second set of animals, aversion to cat odor was quantified in a circular arena congruent to the initial design of reported infection effects. One quadrant contained soiled bedding from home cage of the animal, serving as the home base for exploratory sorties. Cat odor and a novel vanilla odor were placed in two adjoining quadrants. The ratio of time spent in cat quadrant relative to sum time spent in both cat and novel odor quadrants was calculated (chance = 50%). Toxoplasma infection, in contrast to earlier observations in the similar design, reduced percentage time spent near cat urine ( Figure 1C; t _17_ = 2.70, *p* = 0.015). The effect of infection on innate aversion was of robust magnitude (Cohen’s *d* = 1.239; Δ = -16.46% with 95% confidence intervals -29.31 to -3.61%). The maximum of animals from the infected group was again observed to be below the median of the uninfected animals.

Serological examination confirmed that all animals in the infected groups sustained chronic infection with *Toxoplasma gondii*.

### Early life maternal separation stress resulted in anxiolytic behavior in male rats

Animals subjected to early life maternal separation stress were tested in the elevated plus maze and home cage emergence test to determine the effect of maternal separation on anxiety behavior during adulthood.

**Figure 2.**
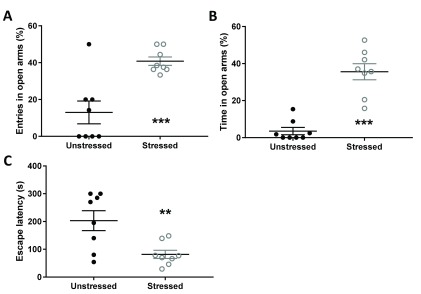
Early life maternal separation stress resulted in increased anxiolytic behavior in male rats. Ordinate depicts number of entries and into the open arm relative to total entries in open and enclosed arms of the elevated plus maze ( **A** and **B**, respectively) and latency to emerge from the home cage into a novel environment ( **C**). Line graphs depict mean and standard error of the mean for unstressed (black) and stressed (gray) male rats. *, *p* < 0.05; **, p < 0.01; unpaired two-tailed Student’s t-test (n = 8 animals in each experimental group).

Stressed animals, in contrast to earlier observations in a similar design, exhibited significantly less anxiety compared to unstressed controls. This was evident as increased percentage entries into anxiogenic open arms of elevated plus-maze ( Figure 2A; t _14_ = 4.21, *p* = 0.0009). Stress-induced anxiolysis was of robust magnitude (Cohen’s *d* = -2.104; Δ = 27.77% with 95% confidence intervals 13.62 to 41.92%). The minimum of animals from the stressed group was higher than all but one animal from the unstressed group. Experimental treatment did not cause significant differences in number of entries made into non-anxiogenic enclosed arms of the maze (t _14_ = 1.98, *p* = 0.07). To preclude effects of entries in enclosed arms on open arm exploration, we further conducted a univariate analysis of variance for percentage open arm entries while employing number of enclosed entries as a covariate. Furthermore, stressed animals spent more time in open arms during the test duration compared to unstressed counterparts ( Figure 2B; t _14_ = 6.69, *p* < 0.001). This analysis revealed a significant increase in open arm exploration due to the stress independent of inter-group differences in enclosed arm entries (F _1,13_ = 14.898, *p* = 0.002). This is congruent with significant increase in number of head dips made during the trial by stressed animals (t _14_ = 3.41, *p* = 0.0042; Δ = 13.25 with 95% confidence intervals 4.94 to 21.56).

Stress-induced anxiolysis was also confirmed by home cage emergence test. In this assay, anxiolysis manifests as reduced latency to emerge into a novel environment from home cage. Stress significantly decreased the latency of home cage emergence ( Figure 2C; t _14_ = 3.14, *p* = 0.0072). Stress-induced anxiolysis was also of robust magnitude in this assay (Cohen’s *d* = -1.57; Δ = -121.4s with 95% confidence intervals -204.2 to -38.5s). The maximum latency of animals from the stressed group was lower than median latency from the unstressed group.

Cat odour avoidance assay Percentage time spent exploring the cat odour stimulus by uninfected and *Toxoplasma*-infected rats in both experiment 1 and 2. Click here for additional data file. Copyright: © 2018 Abdulai-Saiku S et al. 2018 Data associated with the article are available under the terms of the Creative Commons Zero "No rights reserved" data waiver (CC0 1.0 Public domain dedication).

Elevated plus maze anxiety test Escape latency and percentage open arm entries for stressed and unstressed animals. Click here for additional data file. Copyright: © 2018 Abdulai-Saiku S et al. 2018 Data associated with the article are available under the terms of the Creative Commons Zero "No rights reserved" data waiver (CC0 1.0 Public domain dedication).

## Discussion

Experimental treatment in the present report caused robust effects, as evidenced by substantial effect size and clear departure of mean differences from the chance. The direction of these effects is in stark contrast to those observed in previous reports ^1, 6– 8, 20– 22^. For example, multiple experiments in several laboratories indicate that chronic *Toxoplasma gondii* infection causes loss of innate fear to predator odor in male and female rats ^6– 8^. Data in the present report, however, argue for a significant increase in innate fear post-infection. Similarly, stress-induced anxiogenesis has been reported across several laboratories and several paradigms. The current dataset, in contrast, exhibits significant anxiolysis post-stress. The cause of this discrepancy cannot be ascertained with confidence. In fact, we have observed stress-induced anxiogenesis and the infection-induced loss of fear in the same animal facility and same animal strain before these experiments ^1, 5– 7, 13^. The only difference between the experimental circumstances has been a construction project that was ongoing during the present experiments. The construction started across the road from the animal facility after our preceding baselines were conducted and during the present period of the behavioral testing. In fact, we observed reversal to *Toxoplasma*-induced loss of fear in female rats in experiments conducted in the animal facility after the cessation of building construction ^7^.

It remains unclear if the effects of construction related to the change in ambient vibrational environment or some hitherto unknown variable. Although the acoustic noise in frequency range audible to humans remained unchanged during the period, we are not confident that the construction did not change the acoustic environment in sub-audible frequencies. It is interesting that the effects observed here do not correspond to a simplistic notion of greater baseline stress during the period. Effects of stress on anxiety are often presented to have an inverse U kind of reaction norm, whereby increasing stress enhances its effects on the behavioral and health parameters ^23– 26^. We observed an anxiolysis by experimental stress rather than greater anxiogenesis due to the accumulative stress of the treatment and environmental change. Thus, the present observations reiterate the often complex interactions between environment and behavior that could impose significant bounds on the interpretation of laboratory experiments. Related to this, same transgenic mice are known to exhibit divergent behavioral phenotypes across three experimental locations despite careful alignment of experimental protocols ^27^.

Proximate mechanisms of atypical observations in the current study remain unknown, although several possibilities can be posited based on the previous literature. Long-term effects of environment on the behavior often take form of epigenetic modifications in the brain. *Toxoplasma gondii* infection, for example, causes DNA hypomethylation in arginine vasopressin promoter within medial amygdala ^5^. Similarly, maternal separation results in robust hypomethylation in insulin signaling pathway within rat hippocampus ^28^. It is thus plausible that environmental disturbance influenced behavior through epigenetic modifications within the brain. Alternatively alterations in central monoamine levels could also cause the behavioral change. Maternal separation increases monoamine levels within hippocampus and amygdala ^29^ while *Toxoplasma gondii* infection reduced dopamine concentration within nucleus accumbens ^30^. It is plausible that environmental modification changed the nature of monoamine response consequent to the stress or the infection.

## Conclusions

Often, unforeseen changes in the environment near animal facilities can significantly alter the direction of experimental effects in rodent research. This highlights the crucial role of often unreported and unquantified environmental context in the interpretation and replicability of the behavioral data.

## Data availability

The data referenced by this article are under copyright with the following copyright statement: Copyright: © 2018 Abdulai-Saiku S et al.

Data associated with the article are available under the terms of the Creative Commons Zero "No rights reserved" data waiver (CC0 1.0 Public domain dedication). 

**Dataset 1: Cat odour avoidance assay.** Percentage time spent exploring the cat odour stimulus by uninfected and *Toxoplasma*-infected rats in both experiment 1 and 2. 10.5256/f1000research.13171.d186327 ^31^

**Dataset 2: Elevated plus maze anxiety test.** Escape latency and percentage open arm entries for stressed and unstressed animals. 10.5256/f1000research.13171.d186328 ^32^
