# Supplementary material for: Effects of stress or infection on rat behavior show robust reversals due to environmental disturbance
Source: F1000Res. 2018 Jan 16;6:2097. Originally published 2017 Dec 6. [Version 2] doi: 10.12688/f1000research.13171.2 (PMC5782406; doi:10.12688/f1000research.13171.2)
Supplement: Peer review history

# Peer review: Referee response for version 2

**DOI:** 10.5256/f1000research.14793.r29863

**Published:** 2018-01-22

**Authors:** Terence Y. Pang

**Affiliations:** 1 Florey Institute of Neuroscience and Mental Health, University of Melbourne, Parkville, Vic, Australia

The authors have satisfactorily addressed the previous concerns I raised.

I have read this submission. I believe that I have an appropriate level of expertise to confirm that it is of an acceptable scientific standard.

# Peer review: Referee response for version 1

**DOI:** 10.5256/f1000research.14287.r28761

**Published:** 2017-12-19

**Authors:** Terence Y. Pang

**Affiliations:** 1 Florey Institute of Neuroscience and Mental Health, University of Melbourne, Parkville, Vic, Australia

This is a well-written, clearly presented manuscript describing unexpected behavioural phenotypes of two well-established rodent models which routinely lead to rats having anxiolytic or anxiogenic behaviours. In the field of behavioural neuroscience where robustness of results and reproducibility is vital, the reporting of negative or contrary outcomes remains important. This report raises substantial concern for the rodent research occurring across that time period. It is crucial that universities and research institutes be educated on the impact that infrastructure development has on researchers, and the time and financial costs it imposes on research teams/projects.

 In the Introduction, which is rather short, it would be useful to include one or two paragraphs referencing evidence that both Toxoplasma infection and maternal separation models are prone to environmental modification. One of the included references (Koe et al., Transl Psychiatry 2016) is an example of environmental modification of a robust maternal separation-induced adult phenotype. See also Sahafi E et al., Physiol Behav 2018 as another example of an external modifier of anxiety behaviour.

 This manuscript is limited in that there is no molecular data to be paired with the interesting behavioural phenotype. A comparison of monoamine-relevant genes ala Récamier-Carballo S et al., Behav Pharmacol 2017 would have been ideal. But this can be speculated upon in the Discussion. Could also mention the involvement of environment-induced epigenetic changes, see McCoy CR et al., Eur J Neurosci 2016 : DNA methylation changes in the hippocampus.

 The major shortcoming of this manuscript is that I am unsure about how one would go about quantifying structural disturbances. Is it based solely on the unexpected behavioural phenotype observed? Or has the phenotypes consistently shifted during the stated period before returning to “normal”? Have there been anecdotal accounts of construction noise in the rodent facility? Is there any data about building vibrations? (Civil engineers would have the equipment to measure structural vibrations).

 Assuming the significant external source of variability (as compared to a new experimenter who is a inexperienced at handling rodents and conducting the behavioural tests), it would be useful to include litter sizes and M/F sex ratios. Is there body weight data in the event that feeding behaviour was also altered?

 It is unusual to only present EPM data as % entries in open arm. What about total time in open arms as a % of the test duration?

 Is it possible to include schematics of the different test arenas for Figure 1? Do the authors have habituation data (total time spent moving, distance travelled) for odor aversion tests? If the infected rats are anxious, they could be observed to display non- or lesser habituation even at baseline in the absence of a predator odor. If this was the case, it would only serve to strengthen the interpretation.

I have read this submission. I believe that I have an appropriate level of expertise to confirm that it is of an acceptable scientific standard, however I have significant reservations, as outlined above.

# Response

**Published:** 2017-12-27

**Authors:** Rupshi Mitra

**Affiliations:** Nanyang Technological University, Singapore

We thank reviewer for suggestions and comments. This has helped us to improve this manuscript during the revision. We have now submitted version 2 of this manuscript to the F1000Research.

Introduction has been modified in version 2 to include prior work showing that effects of Toxoplasma infection and maternal separation are subject to environmental modification (references 12 through 16 in the bibliography).

We have also revised the discussion to include plausible proximate mechanisms including epigenetic changes and monoamines. Please see paragraphs immediately preceding the conclusions.

We now return to the reviewer’s comment about ambivalent nature of quantifying structural disturbances. We have indeed observed return to stress-induced anxiogenesis and Toxoplasma-induced loss of fear once construction project abated. Toxoplasma effects were eventually published (reference 7 in the revised bibliography; DOI: 10.1016/j.bbi.2017.04.005). Same set of experimenters conducted experiments before, during and after the construction project. Thus, congruent stress- and infection- effects before and after construction project suggest that the environmental modification brought about by the construction explains atypical effects in the interim.

Experimental groups were coded during the experiment. For example, in case of Toxoplasma infection, experimenter did not know infection status of the individual animals; and groups were merely identified with codes during the statistical analysis. Hence we did not notice the reversal until long after the experiment was over, data was analyzed and infection status was confirmed using serology. This precluded systematic investigation of the environmental variables during the period of experiment itself. Although the acoustic noise in frequency range audible to humans remained unchanged during the period, we are not confident that the construction did not change the acoustic environment in sub-audible frequencies. Similarly we did not have opportunity to measure structural vibrations as the project was finished while we were analyzing the data and confirming group assignments using serology.

Toxoplasma gondii infection did not cause significant change in body weight of animals (179.1 ± 4.708, n = 8 for control; 183.1 ± 2.706, n = 7 for infected; p = 0.5). This information is now included in methods section of the revised manuscript. We have revised the manuscript to include date for percentage open arm time ( *p* < 0.001) for EPM in figure 2. We have also included schematics of test arena in revised Figure 1. Please note that this has changed panel number for figures in the results and legends.

Unfortunately, we did not record videos for habituation sessions. We have earlier shown that Toxoplasma infection does not affect locomotion or exploration in open field arena.

# Peer review: Referee response for version 1

**DOI:** 10.5256/f1000research.14287.r28765

**Published:** 2017-12-11

**Authors:** Jaroslav Flegr

**Affiliations:** 1 Department of Biology, Charles University in Prague, Prague, Czech Republic

The authors present interesting data showing that, most likely, an unknown environmental factor or factors can qualitatively modify the behavioral responses of experimental animals on various standard stimuli (here the maternal separation stress and the *Toxoplasma* infection), which could result in unexpected results of standard experiments. The methods are clear and with sufficient details described, and the collected data are analyzed, presented and interpreted in a proper way. The authors suggested that the most probable factor that influenced the outputs of their experiments was (acoustical or mechanical) disturbance from a building construction project that had started adjacent to their animal facility during their experiments. This explanation seems to be reasonable, however, it would be a little bit difficult (and expensive) to test its validity.

 I consider the results (and conclusions) of this study to be not only very interesting, but also very important. It is highly probable that the same or similar phenomena are frequently seen by many researchers, however, they are mostly considered to be just the results of some technical error – “This new student/technician is really terrible, he certainly confused the labels on the cages/test tubes!”. We can just hope that the publication of the present paper will have the “#MeToo effect” – that it will encourage other researchers to publish their own puzzling results.

 I agree that the inverted-U shape (or U-shape) relations between many physiological variables is mostly responsible for frequently observed opposite reaction of a biological system on the same stimuli. Under one situation (e.g., when no building construction project is going on) the background level of stress is low and adding some stress factor, e.g., infecting animals with *Toxoplasma*, will shift the behavioral response toward the maximum of the inverted-U function. Under another situation, when the background level of stress is higher (when the building construction project is going on) additional stress (e.g., the infection with *Toxoplasma*) will shift the behavioral response behind the maximum of the inverted-U function, which will result in an opposite behavioral reaction on the infection. In the review article on the methodological problems of studying the effects of toxoplasmosis using *Toxoplasma*-human model (Flegr, 2013), I showed that on genetically polymorphic outbred animals, including humans, the same factor often influences some individuals in one way and another individuals in an opposite way, depending on their (unknown) genotype. Very often, we can see that population means of the output variable in the affected individual and in the controls remains the same; however, variance of the output variable in the population of affected individuals grows significantly. For example, comparison of Cattell’s 16PF personality profiles of women showed that infected women had higher intelligence and lower guilt proneness than *Toxoplasma*-free women. At the same time, they differed in the variance of four other personality factors, namely protension, surgency, shrewdness and self-sentiment integration (Flegr and Havlíček, 1999). It is therefore very important to study the effects of particular factors not only on population mean of the output variable but also on the variance of this variable. We should never forget that the F-test (or a permutation test performed on squared Z- scores) is not just a pesky technique for testing presumptions of parametric statistical tests, but often it can also be an important and powerful tool for detecting biologically relevant effects of the factor under study.

 Exactly the same mechanism can explain why males and females so often react to the same factor in an opposite way. In most animal species, males and females are not same. Therefore, many physiological parameters of males and females differ in their (mean) position in relation to the maximum of the inverted-U function. Consequently, they will respond to the same factor by the opposite-direction shifts. For example, 10 of 16 Cattell’s personality factors are shifted in an opposite direction in men and women in reaction to the *Toxoplasma* infection (Flegr *et al.*, 2000; Flegr *et al.*, 1996). Similarly, *Toxoplasma*-infected men rate the smell of highly diluted cat urine as more attractive while infected women rate this smell as less attractive than their non-infected peers (Flegr *et al.*, 2011). It is worthwhile in the context of the present Abdyla-Saiku *et al.* article to mention that our recent study showed the very opposite pattern, namely higher attractiveness of the smell in the infected women and lower in the infected men, when undiluted cat urine was used as the stimulus (Flegr *et al.* 2017). 

 Back to the present article. It can be published in its present form. I would just suggest that the authors cite the old study (Vyas *et al.*, 2007) showing the inverted-U shaped response of infected rats on the smell of cat urine. When describing their experimental setup, the authors should better emphasize the fact that stressed mothers, not stressed pubs were used in all ethological tests. Authors should also double-check whether all Latin names of species and genera are printed in italic, both in the main text and in the References.

I have read this submission. I believe that I have an appropriate level of expertise to confirm that it is of an acceptable scientific standard.

## References

[1] FlegrJ: Influence of latent Toxoplasma infection on human personality, physiology and morphology: pros and cons of the Toxoplasma-human model in studying the manipulation hypothesis. *J Exp Biol*.2013;216(Pt 1) : 10.1242/jeb.073635 127-33 10.1242/jeb.073635 23225875

[2] FlegrJHavlícekJ: Changes in the personality profile of young women with latent toxoplasmosis. *Folia Parasitol (Praha)*.1999;46(1) :22-8 10353191

[3] FlegrJKodymPTolarováV: Correlation of duration of latent Toxoplasma gondii infection with personality changes in women. *Biol Psychol*.2000;53(1) :57-68 1087606510.1016/s0301-0511(00)00034-x

[4] FlegrJLenochováPHodnýZVondrováM: Fatal attraction phenomenon in humans: cat odour attractiveness increased for toxoplasma-infected men while decreased for infected women. *PLoS Negl Trop Dis*.2011;5(11) : 10.1371/journal.pntd.0001389 e1389 10.1371/journal.pntd.0001389 22087345PMC3210761

[5] FlegrJZitkováSKodymPFryntaD: Induction of changes in human behaviour by the parasitic protozoan Toxoplasma gondii. *Parasitology*.1996;113 ( Pt 1):49-54 871041410.1017/s0031182000066269

[6] FlegrJMilinskiMKaňkováŠHůlaMHlavačováJSýkorováK: Effects of latent toxoplasmosis on olfactory functions of men and women. *bioRxiv 231795*.2017; Reference source 10.1371/journal.pone.0209773PMC630787130589911

[7] VyasAKimSKSapolskyRM: The effects of toxoplasma infection on rodent behavior are dependent on dose of the stimulus. *Neuroscience*.2007;148(2) : 10.1016/j.neuroscience.2007.06.021 342-8 10.1016/j.neuroscience.2007.06.021 17683872PMC2430144

# Response

**Published:** 2017-12-27

**Authors:** Rupshi Mitra

**Affiliations:** Nanyang Technological University, Singapore

We thank reviewer for suggestions and comments. This has helped us to improve this manuscript during the revision. We have now submitted version 2 of this manuscript to the F1000Research.

In the version 2, we have included a discussion of non-monotonic response of Toxoplasma in the introduction. We would also like to clarify that we tested stressed pups not their mothers. Pups were maternally deprived before weaning, allowed to reach adulthood and then tested.

This has now been made clear during the revision.

We have carefully checked and corrected all Latin names.
